# Association of iron-related parameters with the development of sepsis-associated acute kidney injury

**DOI:** 10.3389/fimmu.2026.1918644

**Published:** 2026-08-26

**Authors:** Pengyi An, Gong Gu, Zhuang Ge, Yingying Dong, Feili Liu, Xuhan Liu, Chuwei Yang

**Affiliations:** 1 Department of Emergency Medicine, The Second Affiliated Hospital of Dalian Medical University, Dalian, China; 2 Department of Emergency Medicine, The People’s Hospital of Liaoning Province, Shenyang, China; 3 Department of Endocrinology, Central Hospital of Dalian University of Technology, Dalian, China; 4 The Second Affiliated Hospital of Dalian Medical University, Dalian, China

**Keywords:** acute kidney injury, association, iron status, sepsis, serum ferritin

## Abstract

**Objective:**

To investigate the associations of iron-related parameters, including serum ferritin (SF), serum iron (SI), and transferrin saturation (TSAT), with the development of sepsis-associated acute kidney injury (SA-AKI).

**Methods:**

This retrospective study included 808 patients with sepsis admitted to the Second Affiliated Hospital of Dalian Medical University between January 1, 2015 and September 30, 2024. Data were obtained from the Yidu Cloud database and the hospital electronic medical record system. Patients were classified according to whether acute kidney injury (AKI) was clinically diagnosed within 48 hours of admission. Group differences were assessed using appropriate parametric or nonparametric tests, and correlations using Spearman analysis. SF, SI, and TSAT were categorized into quartiles to examine trends in AKI incidence. Two multivariable logistic regression models—a clinical model and the clinical model plus SF—were constructed to evaluate factors associated with SA-AKI. Receiver operating characteristic (ROC) curves were used to assess discriminatory ability, and the areas under the curves (AUCs) were compared using the DeLong test. Model calibration was assessed using the Hosmer–Lemeshow test and calibration plots, and internal validation was performed using 1,000 bootstrap resamples.

**Results:**

Among the 808 patients, 310 (38.4%) had SA-AKI. In Model 2, higher SF remained associated with SA-AKI (OR 2.479 per 100-ng/mL increase, 95% CI 2.123,2.894, *P* < 0.001). Adding SF to the clinical model increased the AUC from 0.730 to 0.846 (95% CI 0.085,0.147, *P* < 0.001). Both models showed acceptable calibration, and bootstrap estimates were generally consistent with the original results. For SF alone, the AUC was 0.793 (95% CI 0.761, 0.826), and the optimal cutoff was 525.56 ng/mL, with a sensitivity of 0.710 and a specificity of 0.765.

**Conclusions:**

Higher SF was associated with SA-AKI and improved the discriminatory ability of the clinical model. SF may provide additional information for the clinical assessment and identification of SA-AKI in patients with sepsis.

## 1 Introduction

Because of their high metabolic demand and marked susceptibility to redox imbalance, the kidneys are among the organs most vulnerable to injury during sepsis (1). The development of sepsis-associated acute kidney injury (SA-AKI) often marks a major turning point in the clinical course of sepsis and is closely associated with poor outcomes. A multinational cross-sectional study on acute kidney injury (AKI) epidemiology reported that sepsis is the leading precipitating factor for AKI and that SA-AKI accounts for 40.7% of AKI cases in the intensive care unit (ICU) (2). In addition, a meta-analysis including 189 studies showed that SA-AKI is strongly associated with poor prognosis, with mortality reaching 48%, while approximately 10% of survivors remain dependent on renal replacement therapy (3). At present, AKI is still diagnosed mainly on the basis of serum creatinine (SCr) and urine output. However, both are delayed functional markers and often become abnormal only after substantial kidney injury has already occurred. Identifying earlier and more sensitive parameters therefore remains clinically important.

Increasing evidence suggests that disturbances in iron metabolism are involved in both the onset and progression of sepsis. Inflammatory activation can induce hepcidin overexpression, promoting intracellular iron sequestration and reducing circulating iron availability. Iron deficiency may impair erythropoiesis and oxygen delivery, and may also contribute to multiple organ dysfunction in sepsis through disrupted cellular metabolism and mitochondrial injury (4–7). At the same time, the kidney is a key organ in iron handling, and excess iron deposition may catalyze hydroxyl radical generation, thereby aggravating oxidative stress and lipid peroxidation, ultimately leading to tubular epithelial injury and necrosis (8–10). These processes are central to the pathogenesis of SA-AKI. It is therefore plausible that disordered iron metabolism is not merely a consequence of systemic inflammation in sepsis, but may also directly contribute to renal oxidative injury and cell death.

Several clinical studies have shown that iron-related parameters are associated with prognosis in critically ill patients and in patients with sepsis. Serum ferritin (SF) and serum iron (SI) have been reported to be positively associated with in-hospital mortality in severe sepsis, whereas lower transferrin saturation (TSAT) has been linked to reduced survival (11–13). However, the relationship between iron metabolism and sepsis-associated kidney injury has not been clearly defined.

In this context, the present retrospective study evaluated iron-related parameters measured within 48 hours after admission in patients with sepsis, including SF, SI, and TSAT, and examined their associations with SA-AKI. The findings may provide additional information for clinical assessment and further insight into the role of iron dysregulation in SA-AKI.

## 2 Materials and methods

### 2.1 Data source

This retrospective study used data extracted from the Yidu Cloud electronic medical record system. We included patients who met the diagnostic criteria for sepsis and were admitted to the Second Affiliated Hospital of Dalian Medical University between January 1, 2015 and September 30, 2024. The study was approved by the Ethics Committee of the Second Affiliated Hospital of Dalian Medical University (No. KY2024-242-01).

### 2.2 Selection criteria

Sepsis was defined according to the Third International Consensus Definitions for Sepsis and Septic Shock (Sepsis-3) published in 2016 (14). AKI was diagnosed according to the 2012 Kidney Disease: Improving Global Outcomes (KDIGO) Clinical Practice Guideline for AKI (15).

The inclusion criteria were as follows: (1) age ≥18 years; (2) diagnosis of sepsis based on the Sepsis-3 criteria; and (3) completion of the laboratory tests required for this study within 48 hours after admission.

**Figure 1:**
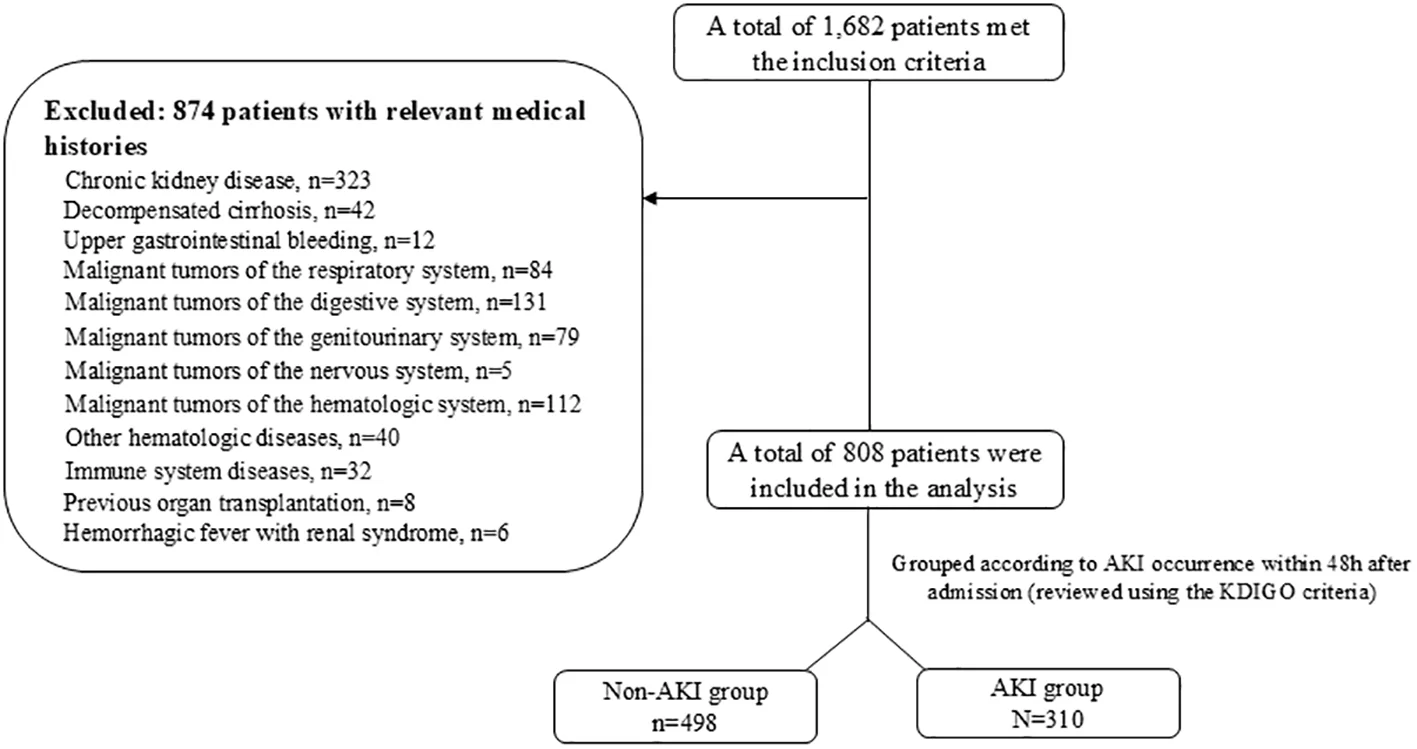
Flowchart of patient selection and grouping

The exclusion criteria were as follows: (1) chronic kidney disease; (2) decompensated cirrhosis; (3) upper gastrointestinal bleeding; (4) malignant tumors; (5) hematologic diseases; (6) immune disorders; (7) prior organ transplantation; (8) other diseases associated with kidney injury; and (9) long-term regular use before admission or ongoing use at admission of medications that might affect renal function or iron metabolism (**Figure 1**).

When a preadmission SCr value was available in the electronic medical records, it was used as the baseline SCr. Patients with documented pre-existing abnormal SCr values, chronic kidney disease, or other known kidney diseases were excluded. When no historical SCr value was available, the sex-specific upper limit of the institutional laboratory reference range was used as the surrogate baseline SCr value: 104 μmol/L for adult males and 84 μmol/L for adult females, as measured using an enzymatic method.

### 2.3 Data collection

The following baseline variables were collected: age, sex, medical history, infection site at admission (respiratory, urinary, abdominal, or other), mean arterial pressure (MAP), and Sequential Organ Failure Assessment (SOFA) score.

Laboratory results obtained within 48 hours after admission were also collected, including routine blood indices [white blood cell count (WBC), neutrophil count (NEU), platelet count (PLT), red blood cell count (RBC), and hemoglobin (Hb)]; inflammatory markers [C-reactive protein (CRP) and procalcitonin (PCT)]; liver function indices [total bilirubin (TBil), alanine aminotransferase (ALT), and aspartate aminotransferase (AST)]; renal function indices [SCr and blood urea nitrogen (BUN)]; iron-related parameters [SF, SI, and TSAT]; tissue perfusion marker [lactate (LAC)]; and coagulation parameters [D-dimer, prothrombin time activity (PTA), fibrinogen (Fbg), and activated partial thromboplastin time (APTT)].

### 2.4 Study outcome

Patients were initially classified into the non-AKI and AKI groups according to whether AKI had been clinically diagnosed and documented in the medical records within the first 48 hours after admission. All patients classified as having AKI were subsequently reviewed by the investigators to confirm that they met the KDIGO SCr criteria for AKI.

### 2.5 Statistical analysis

Statistical analyses were performed using IBM SPSS Statistics version 26.0. Continuous variables were assessed for normality. Normally distributed variables are presented as the mean ± standard deviation and were compared using the independent-samples t test. Non-normally distributed variables are presented as the median (Q1, Q3) and were compared using the Mann–Whitney U test. Categorical variables are presented as numbers and percentages [n (%)] and were compared using the chi-square test.

Spearman correlation analysis was used to assess the associations between iron-related parameters and SA-AKI. SF, SI, and TSAT were further categorized into quartiles to examine trends in the incidence of SA-AKI across increasing quartiles.

Variables included in the clinical model were selected according to clinical relevance and data availability. The linearity of continuous variables in the logit was assessed using the Box–Tidwell test. To account for multiple testing, a Bonferroni-adjusted significance threshold, calculated as 0.05 divided by the number of continuous variables examined, was applied. Continuous variables that did not satisfy the linearity assumption were natural-log transformed and reassessed before model fitting.

Two multivariable logistic regression models were subsequently constructed to evaluate factors associated with SA-AKI. Model 1 included age, sex, hypertension, diabetes mellitus, coronary artery disease, stroke, infection-site indicators, WBC, PLT, PCT, LAC, D-dimer, and MAP. Model 2 included all variables in Model 1 with the addition of SF. Multicollinearity among the variables included in each model was assessed using the variance inflation factor (VIF), with a VIF <5 considered to indicate the absence of substantial multicollinearity. Logistic regression results are reported as ORs, 95% CI, and corresponding *P* values.

Receiver operating characteristic (ROC) curves were constructed, and the areas under the curves (AUCs) with 95% CIs were calculated to assess the discriminatory ability of the iron-related parameters and the two multivariable models for identifying SA-AKI. The ROC curves for Model 1 and Model 2 were generated using the predicted probabilities derived from the corresponding models. The optimal cutoff values for the iron-related parameters were determined using the maximum Youden index. The AUCs of Model 1 and Model 2 were compared using the DeLong test.

Model calibration was evaluated using the Hosmer–Lemeshow goodness-of-fit test and calibration plots comparing observed and predicted probabilities across deciles of predicted risk. Internal validation was performed using bootstrap resampling with 1,000 repetitions to assess the stability of the regression estimates. All statistical tests were two-sided, and a *P* < 0.05 was considered statistically significant, except when a Bonferroni-adjusted significance threshold was applied.

## 3 Results

### 3.1 Patient characteristics

A total of 808 patients with sepsis were included, of whom 498 (61.6%) were assigned to the non-AKI group and 310 (38.4%) to the AKI group. Among the iron-related parameters, SF was significantly higher in the AKI group, whereas SI and TSAT were significantly lower (all *P* < 0.05) (**Table 1**).

**Table 1:** Comparison of baseline clinical characteristics between the SA-AKI and non-SA-AKI groups.

Variable	Non-AKI group (n=498)	AKI group (n=310)	*Z*/*χ*^2^/*t*	*P*
Age	76 (65, 83)	78 (69, 84)	-1.921	0.055
Sex				
Male	269 (54.0%)	170 (54.8%)		
Female	229 (46.0%)	140 (45.2%)		
Comorbidities				
Hypertension	251 (50.4%)	146 (47.1%)	0.835	0.361
Diabetes mellitus	179 (35.9%)	125 (40.3%)	1.561	0.212
Coronary artery disease	134 (26.9%)	101 (32.6%)	2.981	0.084
Stroke	147 (29.5%)	77 (24.8%)	2.088	0.148
None	118 (23.7%)	68 (21.9%)	0.334	0.563
Site of infection				
Respiratory tract	372 (74.7%)	220 (71.0%)	1.358	0.244
Urinary tract	230 (46.2%)	153 (49.4%)	0.770	0.380
Intra-abdominal	77 (15.5%)	35 (11.3%)	2.785	0.095
Other	34 (6.8%)	20 (6.5%)	0.043	0.835
Multiple sites	195 (39.2%)	111 (35.8%)	0.911	0.340
SOFA (score)	7.03 ± 3.11	8.40 ± 3.56	-5.604	<0.001
WBC (×10^9^/L)	11.32 (7.84,15.16)	12.95 (10.09,17.12)	-4.539	<0.001
NEU (×10^9^/L)	9.59 (6.12,13.44)	11.11 (8.19,15.42)	-4.492	<0.001
PLT (×10^9^/L)	198 (139,264)	175 (111,245)	-3.221	0.001
RBC (×10^12^/L)	3.94 ± 0.70	3.58 ± 0.91	5.918	<0.001
Hb (g/L)	117.70 ± 22.58	105.33 ± 29.44	6.334	<0.001
CRP (mg/L)	81.29 (29.10,148.28)	114.50 (40.47,199.15)	-3.651	<0.001
PCT (ng/mL)	0.46 (0.11,2.49)	2.36 (0.41,12.04)	-8.507	<0.001
TBil (μmol/L)	15.6 (9.71, 22.46)	14.75 (10.58, 20.61)	-0.318	0.750
ALT (U/L)	22.08 (13.00,38.46)	24.25 (14.00,46.83)	-1.434	0.152
AST (U/L)	25.50 (18.20,45.35)	30.07 (18.00,67.85)	-2.744	0.006
SCr (μmol/L)	62.95 (50.06,75.50)	194.69 (142.93,256.27)	-23.766	<0.001
BUN (mmol/L)	6.00 (4.40,8.32)	17.60 (12.35,25.12)	-20.398	<0.001
SF (ng/mL)	449.36 (377.85,522.76)	615.14 (512.22,713.02)	-14.043	<0.001
SI (μmol/L)	13.37 (9.35,19.81)	8.43 (4.69,13.65)	-10.127	<0.001
TSAT (%)	33.08 (24.52,43.41)	20.56 (14.35,34.29)	-9.277	<0.001
LAC (mmol/L)	1.79 (1.38,2.66)	1.70 (1.34,2.79)	-0.724	0.469
D-dimer (μg/mL)	1.69 (1.02,3.36)	2.64 (1.54,6.26)	-7.175	<0.001
PTA (%)	82.36 ± 19.24	76.32 ± 18.77	4.379	<0.001
Fbg (g/L)	5.02 ± 1.97	5.00 ± 2.04	0.083	0.934
APTT (s)	39.30 (34.90,44.63)	39.90 (35.10,46.13)	-1.353	0.176
MAP (mmHg)	93.94 ± 17.44	92.42 ± 19.73	1.115	0.265

Other mainly included hematologic and lymphatic system infections, nervous system infections, bone and joint infections, and skin and soft tissue infections.

### 3.2 Correlations between iron-related parameters and SA-AKI

Spearman correlation analysis showed that SF was positively correlated with AKI occurrence, whereas SI and TSAT were moderately negatively correlated with AKI occurrence. All correlations were statistically significant (all *P* < 0.05) (**Table 2**).

**Table 2:** Associations of iron-related parameters with the risk of SA-AKI.

Variable	*r*	95% CI	*P*
SF	0.494	0.439, 0.546	<0.001
SI	-0.356	-0.417, -0.293	<0.001
TSAT	-0.327	-0.389, -0.262	<0.001

### 3.3 Associations of iron-related parameters with SA-AKI incidence

According to the study by Sun et al. (16), patients were stratified into quartiles based on baseline SF, SI, and TSAT levels. The incidence of AKI increased progressively across SF quartiles (*P* < 0.001). In contrast, AKI incidence decreased progressively across SI quartiles (*P* < 0.001). A similar overall trend was observed for TSAT (*P* < 0.001), although the difference between Q3 and Q4 was not statistically significant (*P* = 0.195) (**Table 3**).

**Table 3:** Proportions of SA-AKI across quartiles of iron-related parameters.

Variable	Q1	Q2	Q3	Q4	*P*
SF	25 (12.4%)	44 (21.8%)	85 (42.1%)	156 (77.2%)	<0.001
SI	143 (70.8%)	62 (30.7%)	60 (29.7%)	45 (22.3%)	<0.001
TSAT	142 (70.3%)	67 (33.2%)	49 (24.3%)	52 (25.7%)^#^	<0.001

# *P* = 0.195 for the comparison between the Q3 and Q4 groups.

### 3.4 Multivariable logistic regression analyses

The Box–Tidwell assessment indicated that PCT required natural-log transformation. After transformation, the continuous variables included in the final models satisfied the linearity assumption in the logit according to the Bonferroni-adjusted significance threshold. No substantial multicollinearity was detected in either model, with all VIF values below 5.

In Model 1, age, stroke, intra-abdominal infection, WBC, natural-log-transformed PCT, and D-dimer were significantly associated with SA-AKI. After SF was added to the clinical model, SF was significantly associated with SA-AKI, with an OR of 2.479 per 100-ng/mL increase (95% CI 2.123,2.894, *P* < 0.001). Age, stroke, respiratory tract infection, intra-abdominal infection, natural-log-transformed PCT, and D-dimer also remained significantly associated with SA-AKI in Model 2, whereas WBC no longer reached statistical significance (P = 0.066) (**Table 4**).

**Table 4:** Multivariable logistic regression analyses of the clinical model and the clinical model plus SF for SA-AKI.

Variable	Model 1			Model 2		
	OR	95% CI	*P*	OR	95% CI	*P*
Age	1.018	1.005,1.030	0.005	1.016	1.002,1.031	0.027
Sex	1.106	0.797,1.537	0.547	1.077	0.739,1.569	0.700
Comorbidities						
Hypertension	0.913	0.650,1.283	0.601	1.132	0.769,1.667	0.530
Diabetes mellitus	1.100	0.786,1.537	0.579	0.869	0.594,1.271	0.469
Coronary artery disease	1.277	0.905,1.803	0.164	1.110	0.748,1.647	0.603
Stroke	0.625	0.434,0.902	0.012	0.572	0.377,0.869	0.009
Site of infection						
Respiratory tract	0.756	0.503,1.135	0.177	0.609	0.382,0.970	0.037
Urinary tract	1.239	0.872,1.759	0.232	1.423	0.953,2.125	0.084
Intra-abdominal	0.390	0.231,0.658	<0.001	0.378	0.210,0.682	0.001
Other	0.780	0.408,1.492	0.452	0.695	0.329,1.466	0.339
WBC (×10^9^/L)	1.039	1.012,1.066	0.004	1.027	0.998,1.056	0.066
PLT (×10^9^/L)	0.999	0.998,1.001	0.307	1.000	0.998,1.002	0.886
ln[PCT (ng/mL)]	1.336	1.234,1.448	<0.001	1.339	1.223,1.466	<0.001
LAC (mmol/L)	1.006	0.938,1.080	0.862	0.981	0.907,1.061	0.632
D-dimer (μg/mL)	1.072	1.036,1.110	<0.001	1.064	1.024,1.104	0.001
MAP (mmHg)	1.001	0.993,1.010	0.750	1.004	0.995,1.014	0.380
SF (per 100 ng/mL)				2.479	2.123,2.894	<0.001

PCT was natural-log transformed to satisfy the linearity assumption in the logit. The OR for SF was expressed per 100-ng/mL increase. For binary variables, the reference categories were male for sex and absence of the corresponding comorbidity or infection-site indicator.

### 3.5 Discrimination, calibration, and internal validation of the models

**Figure 2:**
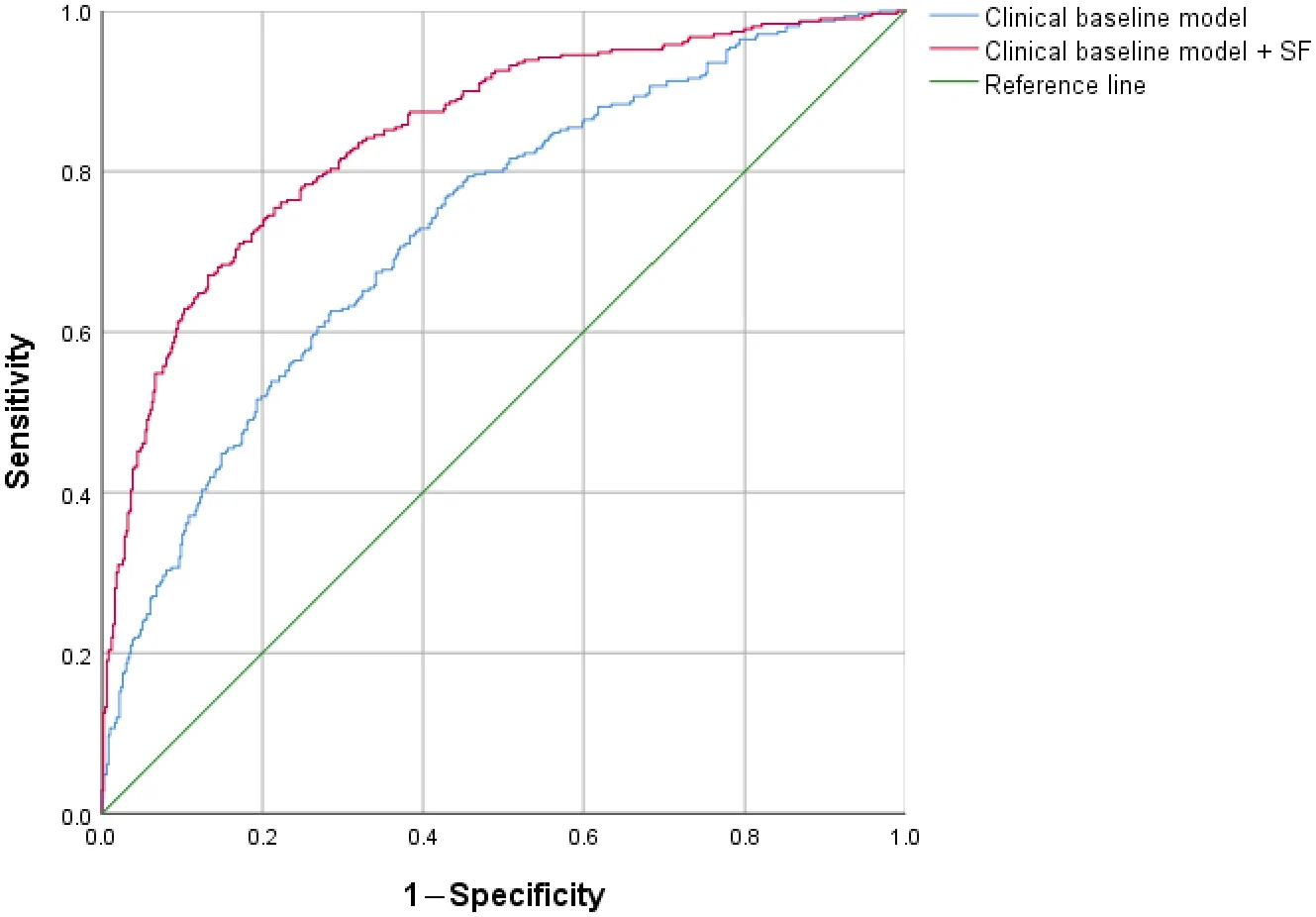
Receiver operating characteristic curves of model 1 and model 2 for identifying SA-AKI.

Model 1 showed an AUC of 0.730 (95% CI 0.695,0.766, *P* < 0.001), whereas Model 2 showed an AUC of 0.846 (95% CI 0.818,0.874, *P* < 0.001). The AUC of Model 2 was 0.116 higher than that of Model 1 (95% CI 0.085,0.147), and the difference was statistically significant according to the DeLong test (*P* < 0.001), indicating improved discrimination after the addition of SF (**Table 5**; **Figure 2**).

**Table 5:** Discrimination and calibration of the two multivariable models for SA-AKI.

Model	AUC	95% CI	Hosmer–Lemeshow χ^2^	df	*P*
Model 1	0.730	0.695, 0.766	4.854	8	0.773
Model 2	0.846	0.818, 0.874	9.400	8	0.310

The difference in AUC between Model 2 and Model 1 was 0.116 (95% CI 0.085–0.147; P<0.001), as assessed using the DeLong test.

**Figure 3:**
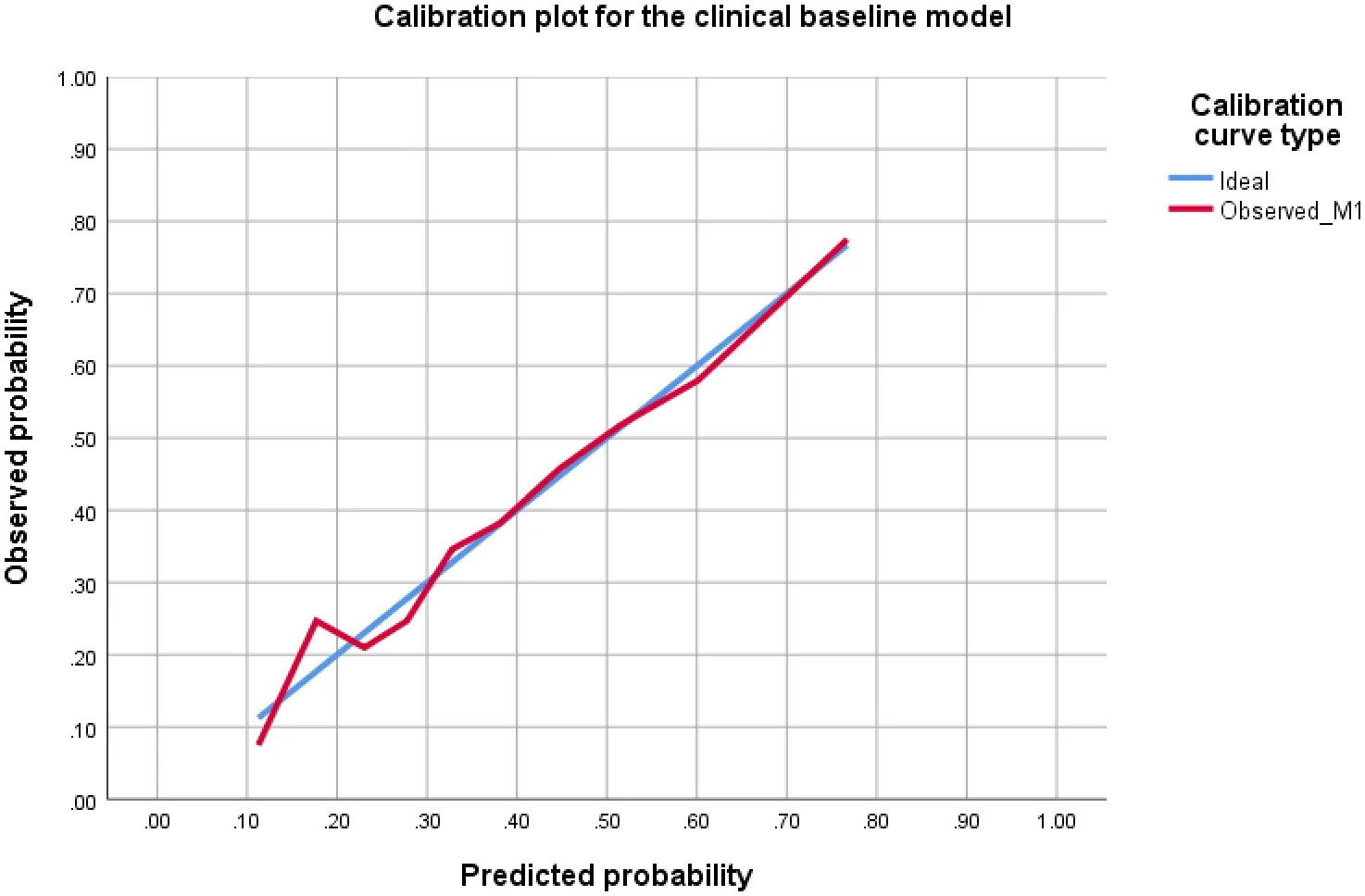
Calibration plot for model 1. The diagonal line represents perfect calibration, and the observed line represents the agreement between predicted and observed probabilities.

**Figure 4:**
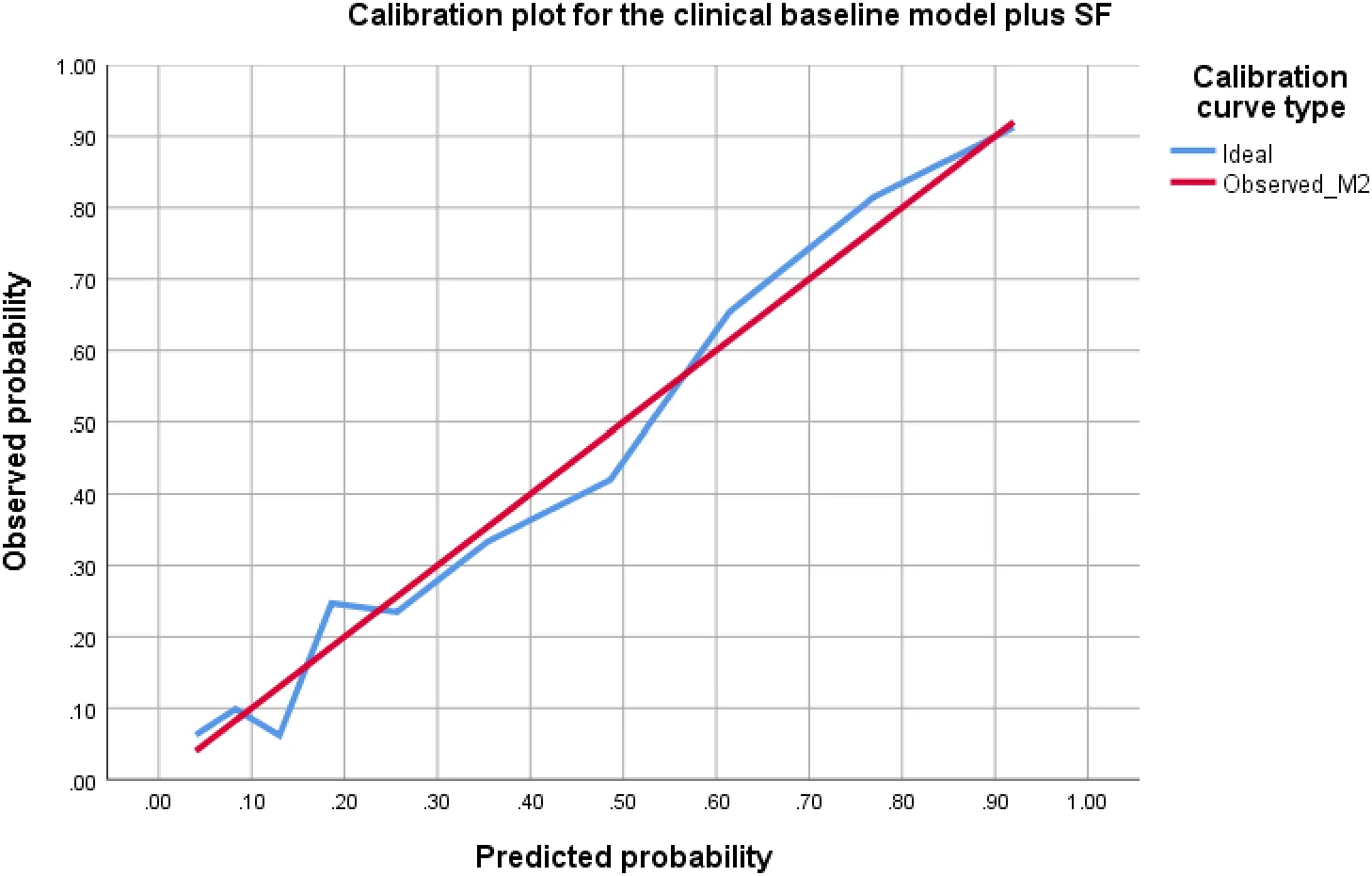
Calibration plot for model 2. The diagonal line represents perfect calibration, and the observed line represents the agreement between predicted and observed probabilities.

The Hosmer–Lemeshow tests showed no evidence of poor fit for either Model 1 (χ²=4.854, df=8, *P* = 0.773) or Model 2 (χ²=9.400, df=8, *P* = 0.310). The calibration plots showed generally close agreement between the observed and predicted probabilities in both models (**Figures 3**, **4**).

Bootstrap internal validation with 1,000 resamples yielded regression estimates that were generally consistent with those of the original models. In Model 2, the association between SF and SA-AKI remained statistically significant after bootstrap validation (B = 0.908, BCa 95% CI 0.717,1.195, *P* = 0.001) (**Supplementary Table 1**).

### 3.6 Discriminatory ability of SF for identifying SA-AKI

**Figure 5:**
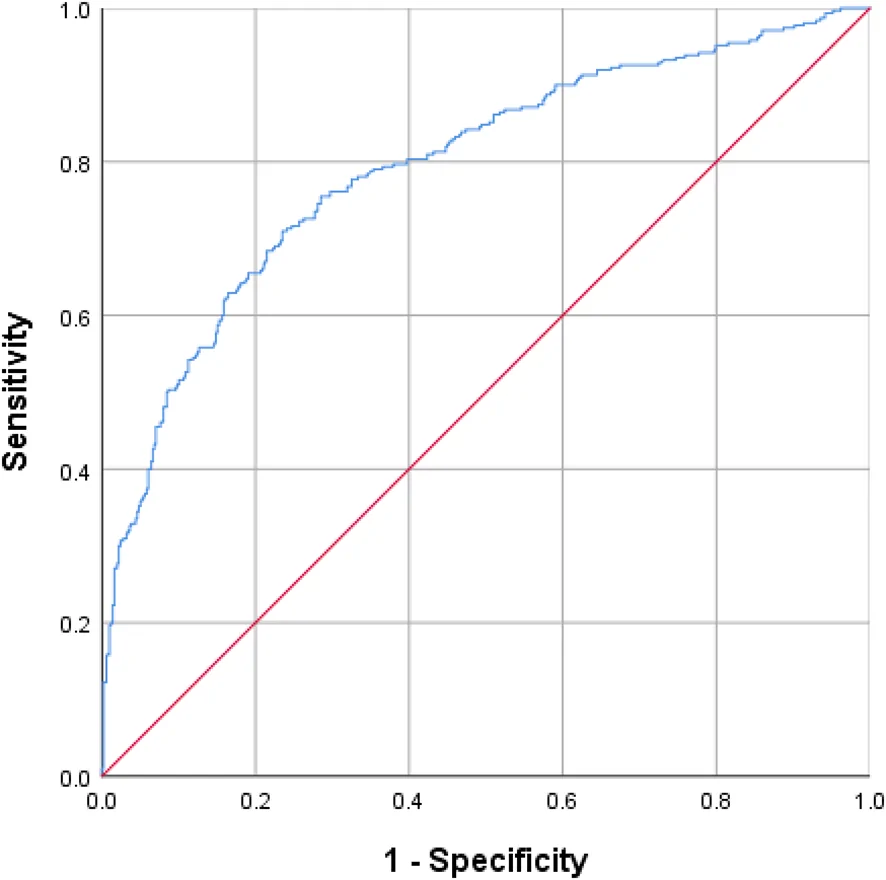
ROC curve of SF for identifying SA-AKI.

ROC curve analysis showed that SF had good discriminatory ability for identifying SA-AKI, with an AUC of 0.793 (95% CI 0.761–0.826, P<0.001). The optimal cutoff value determined using the maximum Youden index was 525.56 ng/mL, with a sensitivity of 0.710 and a specificity of 0.765. (**Table 6**; **Figure 5**).

**Table 6:** Discriminatory performance of SF for identifying SA-AKI.

Variable	AUC	95% CI	Sensitivity	Specificity	Cut-off value	*P*
SF	0.793	0.761, 0.826	0.710	0.765	525.56 ng/mL	<0.001

## 4 Discussion

Sepsis is one of the leading causes of AKI in critically ill patients, and approximately 40%–50% of AKI cases are associated with sepsis or septic shock (17–20). A recent meta-analysis including 189 studies showed that the occurrence of SA-AKI significantly increases the risk of death in patients with sepsis, with mortality reaching as high as 48%. Even among survivors, the proportion of patients left with persistent renal dysfunction may be as high as 40% (3). However, in current clinical practice, the diagnosis of AKI still relies mainly on changes in SCr and urine output. Both markers usually become abnormal only after renal function has already been substantially impaired, making them insufficiently sensitive for timely detection of early renal parenchymal injury and potentially delaying the optimal window for intervention. Given the complex pathogenesis and rapid progression of SA-AKI, identifying clinically accessible parameters associated with SA-AKI may provide additional information for clinical assessment and risk stratification in patients with sepsis. Against this background, the present study systematically examined the associations between iron-related parameters measured within 48 hours after admission and SA-AKI.

In the present study, the incidence of SA-AKI was 38.4%, which is comparable to recent epidemiological reports on SA-AKI. These findings further indicate that SA-AKI remains one of the most common complications in patients with sepsis. To some extent, they also support the representativeness of our study population and the reliability of the subsequent analyses, thereby providing a reasonable epidemiological basis for exploring the relationship between iron metabolism and SA-AKI.

A substantial body of evidence has shown that alterations in iron metabolism are closely related to the development of sepsis and to adverse outcomes. Inflammation-induced elevation of hepcidin promotes intracellular iron sequestration and reduces circulating iron availability. Disturbance of iron metabolism is considered an important contributor to the occurrence of sepsis and to poor prognosis (4, 5, 7). A meta-analysis including 154 randomized controlled trials involving 32,920 participants showed that, compared with oral iron supplementation or no iron supplementation, intravenous iron administration was associated with an increased risk of infection (RR 1.17, 95% CI 1.04, 1.31) (21). Because of the limited number of eligible studies, however, that meta-analysis was unable to compare the effects of oral iron supplementation with those of no supplementation. In an observational study on iron status and sepsis, Hamilton F et al. also found that higher levels of iron parameters were associated with increased odds of sepsis, with odds ratios of 1.17 (95% CI 1.04, 1.29) for SF, 1.13 (95% CI 1.03, 1.23) for SI, and 1.17 (95% CI 1.09, 1.27) for TSAT (6). In addition, SF has been associated with adverse outcomes in patients with sepsis. In a study of 2,451 patients with sepsis, Fang YP et al. showed that SF was linearly positively associated with in-hospital mortality, and that patients with elevated ferritin had a higher risk of death during hospitalization (11).

As an important organ in iron metabolism, the kidney is involved in iron recovery and reutilization, but is also vulnerable to the effects of disturbed iron homeostasis. Maintenance of iron homeostasis is critical to renal cell function and survival (8, 9). Under physiological conditions, renal tubular epithelial cells (RTECs) take up iron through transferrin receptors (TfR) and store it in ferritin to prevent the toxic effects of free iron (10). Under inflammatory conditions, inflammatory mediators induce upregulation of hepcidin, which suppresses the expression of the iron exporter ferroportin and leads to intracellular iron retention. Increased levels of free iron in the kidney can generate excessive reactive oxygen species (ROS) through the Fenton reaction, triggering lipid peroxidation and mitochondrial injury and ultimately causing RTEC dysfunction or death, which is considered one of the key mechanisms in AKI (22). At the same time, inflammation and ischemia-reperfusion injury can impair ion transport in RTECs, further aggravating iron dysregulation. Subsequent iron accumulation in the kidney amplifies oxidative stress, creating a vicious cycle that worsens renal injury. Previous animal studies have provided mechanistic support for the pathological role of disturbed iron metabolism in SA-AKI. In a murine model of sepsis, Xiao J et al. observed marked disruption of iron homeostasis during SA-AKI and further showed that activation of Nrf2, a key regulator of iron metabolism, alleviated renal injury, possibly by improving iron metabolic disturbances (23).

Ferritin is an important iron storage protein and is generally regarded as a reliable biomarker of body iron stores under non-inflammatory conditions. During inflammation, however, ferritin is involved not only in iron homeostasis but also in oxidative stress. Previous studies have shown that elevated SF under inflammatory conditions may be accompanied by activation of the ferritinophagy regulator NCOA4, which promotes ferritin degradation and increases intracellular free iron, thereby aggravating oxidative stress and mediating tubular injury (24). Animal experiments by Jin L et al. further confirmed that modulation of the NCOA4-related pathway can reduce renal iron accumulation and lipid peroxidation, thereby attenuating kidney injury (25).

Taken together, these studies provide mechanistic support for a potential role of ferritin and ferritin-regulated iron metabolic pathways in the pathogenesis of SA-AKI. Although existing studies collectively suggest that iron may be involved in sepsis-related kidney injury, clinical studies directly examining the association between iron metabolism and SA-AKI remain limited. In the present study, comparison of the AKI and non-AKI groups showed significant differences in iron-related parameters. Specifically, SF was higher in the AKI group, whereas SI and TSAT were lower; all differences were statistically significant (all *P* < 0.05). These findings indicate that iron metabolic status differs between patients with and without SA-AKI and provide preliminary clinical evidence of an association between iron dysregulation and SA-AKI. Further correlation analysis showed that SF was moderately positively correlated with SA-AKI status (*r* = 0.494, 95% CI 0.439, 0.546), suggesting that higher SF levels measured within 48 hours after admission were associated with SA-AKI.

However, previous studies have not reached consistent conclusions regarding the role of ferritin in SA-AKI. One animal study found that ferritin heavy chain (FtH)-deficient mice had markedly increased SF levels but a lower incidence of SA-AKI and higher survival (26). In addition, a prospective cohort study showed that higher SF levels at admission were associated with better renal recovery in patients with AKI (27). These apparently discrepant findings suggest that, given the complexity of iron metabolism in SA-AKI, the biological significance of ferritin may not be determined by its circulating level alone. A more comprehensive assessment incorporating other iron-related parameters, such as SI and TSAT, may therefore help characterize the overall state of iron dysregulation.

In sepsis, inflammatory responses can suppress iron export through hepcidin-mediated pathways, leading to reduced circulating iron levels. Clinically, this is often reflected by lower SI and TSAT, which is consistent with the typical features of anemia of inflammation (28–30). In line with these pathophysiological characteristics, our results showed that both SI and TSAT were moderately negatively correlated with SA-AKI status, with correlation coefficients of -0.356 (95% CI -0.417, -0.293) and -0.327 (95% CI -0.389, -0.262), respectively. These findings suggest that reduced circulating iron levels and lower iron availability may be associated with SA-AKI. They are also consistent with the possibility that iron dysregulation in inflammatory states may contribute to renal injury by aggravating oxidative stress or disrupting energy metabolism.

It should also be noted that previous studies examining the relationships of SI and TSAT with infection and sepsis outcomes have yielded inconsistent results. Although no clinical study has directly investigated the associations of SI and TSAT with SA-AKI, the study by Hamilton F et al. showed that higher SI and TSAT were positively associated with the risk of sepsis (6). By contrast, a prospective Norwegian study found that participants with SI at or below the 2.5th percentile (HR 1.72, 95% CI 1.34, 2.21) or TSAT at or below the 2.5th percentile (HR 1.48, 95% CI 1.12, 1.96) had an increased risk of bloodstream infection (31). These differences suggest that the clinical significance of SI and TSAT in inflammatory states may vary according to the clinical context rather than follow a simple linear pattern. One possible explanation is that SI and TSAT show marked dynamic fluctuations during acute inflammation and are readily influenced by the severity of inflammation and by clinical interventions. Consequently, the associations observed for SI and TSAT may differ across studies, limiting their stability and discriminatory performance as clinical assessment markers.

To further examine whether the observed associations were consistent across different levels of the iron-related parameters, we performed trend analyses using quartiles of iron-related parameters. The results showed that, across SF quartiles, the proportion of patients with SA-AKI increased progressively with increasing SF levels. By contrast, in both the SI and TSAT quartile analyses, the overall proportion of SA-AKI decreased as biomarker levels increased, and all of these trends were statistically significant (all *P* < 0.001). In the TSAT quartile analysis, there was some fluctuation in the proportion of SA-AKI between Q3 and Q4, but the difference did not reach statistical significance (*P* = 0.195). This finding may reflect sampling variation or heterogeneity in inflammatory status within the higher TSAT range and should therefore be interpreted cautiously. Overall, the quartile analyses were consistent in direction with the correlation analyses and further supported an association between iron dysregulation and SA-AKI.

To further examine whether SF was associated with SA-AKI after adjustment for clinical factors, we constructed a clinical model and then added SF to this model. In Model 2, higher SF remained independently associated with SA-AKI, with an OR of 2.479 per 100-ng/mL increase (95% CI 2.123–2.894, P<0.001). Adding SF increased the AUC from 0.730 to 0.846, and bootstrap validation yielded generally consistent estimates. These findings suggest that SF may provide additional information on SA-AKI beyond the clinical variables included in the baseline model. In addition, a previous study in transfusion-dependent pediatric patients with thalassemia reported an opposite pattern: starting from a SF level of 1250 μg/L, each decrease of 250 μg/L was associated with an increased risk of AKI (OR 1.25, 95% CI 1.01, 1.56) (32). This finding differs from ours, but the study population was substantially different. That study focused on chronically transfusion-dependent children, in whom long-term iron overload and iron chelation therapy may dominate both ferritin variation and the pattern of renal injury. By contrast, the present study focused on SF measured within 48 hours after admission in adults with sepsis, in whom elevated SF under acute inflammatory conditions may reflect inflammation-related iron sequestration and the accompanying renal injury. The discrepancy between these studies therefore suggests that the clinical significance of ferritin may differ across disease stages and patient populations. Taken together with our findings, higher SF was independently associated with SA-AKI in patients with sepsis and improved the discriminatory ability of the clinical model.

To assess the additional value of SF in the clinical model, we compared the discriminatory performance of Model 1 and Model 2. The AUC increased from 0.730 for Model 1 to 0.846 for Model 2, with an AUC difference of 0.116 (95% CI 0.085–0.147; P<0.001 by the DeLong test). Both models showed acceptable calibration, and the bootstrap estimates were generally consistent with the original results. These findings indicate that adding SF improved the discriminatory ability of the clinical model.

To further evaluate the discriminatory ability of SF alone for identifying SA-AKI, we performed ROC curve analysis. The results showed that SF demonstrated good discriminatory ability for identifying SA-AKI (AUC = 0.793, 95% CI 0.761–0.826, P<0.001), with an optimal cutoff value of 525.56 ng/mL, a sensitivity of 0.710, and a specificity of 0.765. By comparison, in the study by Fang YP et al., which examined in-hospital mortality in patients with sepsis, SF ≥591.5 ng/mL was identified as an independent predictor of death, with an AUC of 0.651 (11). In pediatric chronic disease populations, the reported cutoff was even higher, at 1250 μg/L. These differences in cutoff values may be attributable to several factors. First, the study endpoints differed: the present study focused on identifying SA-AKI, whereas mortality studies and studies conducted in chronic iron-overload settings reflect longer-term or more severe adverse outcomes. Second, the timing of measurement and inflammatory status may differ substantially across studies. SF may rise sharply during acute inflammation, whereas chronic iron overload or iron chelation therapy may lead to higher baseline ferritin levels. Third, differences in study population and assay methods may also affect the determination of the optimal threshold. Overall, although the specific cutoff values differ, these studies consistently suggest that elevated SF may have clinical relevance across different patient populations and outcomes, but the applicable threshold may vary according to the clinical setting.

In summary, the present study systematically evaluated the associations between iron-related parameters and SA-AKI. Elevated SF was significantly associated with SA-AKI, whereas SI and TSAT were lower in patients with SA-AKI, suggesting the presence of disturbed iron metabolism. The addition of SF improved the discriminatory ability of the clinical model, and the association between SF and SA-AKI remained stable in the bootstrap analysis. These findings indicate that SF may serve as a useful marker for identifying SA-AKI and may provide additional information for clinical assessment and risk stratification. This study also has several limitations. First, this was a single-center retrospective study, and laboratory data were unavailable for some patients. Second, although both iron-related parameters and AKI were assessed within the first 48 hours after admission, their exact temporal sequence could not be determined in every patient. Therefore, some patients may already have met the diagnostic criteria for AKI when iron-related parameters were measured, which may have affected the interpretation of the observed associations and the discriminatory ability of SF. Third, baseline SCr could not be accurately determined in all patients, and the use of a surrogate baseline in some cases may have affected AKI classification, potentially leading to underdiagnosis or overdiagnosis of AKI. Fourth, only three iron-related parameters were analyzed, and dynamic changes in iron homeostasis could not be assessed. Future multicenter prospective studies with standardized assessment of baseline kidney function, serial measurements of iron-related parameters, and accurate documentation of AKI onset are needed to further clarify the relationship between iron metabolism and SA-AKI and to validate the clinical significance of SF. In addition, further studies are needed to determine whether interventions targeting iron metabolism could have a role in the prevention or treatment of SA-AKI.

## Data Availability

The raw data supporting the conclusions of this article will be made available by the authors, without undue reservation.
