# Supplementary material for: Association of iron-related parameters with the development of sepsis-associated acute kidney injury
Source: Front Immunol. 2026 Aug 26;17:1918644. doi: 10.3389/fimmu.2026.1918644 (PMC13553410; doi:10.3389/fimmu.2026.1918644)
Supplement: Supplementary file 1 [file Table1.docx]

Supplementary Table S1. Bootstrap internal validation of the multivariable logistic regression models for SA-AKI

Model 1: Clinical baseline model

| Variable | Bootstrap | | | | |
| --- | --- | --- | --- | --- | --- |
|  | B | Bias | SE | *P* | BCa 95% CI |
| Age | 0.017 | 0.000 | 0.007 | 0.011 | 0.004,0.031 |
| Sex | 0.101 | −0.001 | 0.171 | 0.536 | −0.228,0.449 |
| Comorbidities |  |  |  |  |  |
| Hypertension | -0.091 | 0.003 | 0.181 | 0.603 | −0.434, 0.275 |
| Diabetes mellitus | 0.095 | 0.006 | 0.179 | 0.578 | −0.251, 0.486 |
| Coronary artery disease | 0.245 | 0.006 | 0.185 | 0.180 | −0.144, 0.633 |
| Stroke | -0.469 | −0.015 | 0.195 | 0.016 | −0.849,−0.128 |
| Site of infection |  |  |  |  |  |
| Respiratory tract | -0.280 | 0.000 | 0.216 | 0.182 | −0.686,0.135 |
| Urinary tract | 0.214 | 0.017 | 0.188 | 0.243 | −0.185,0.621 |
| Intra-abdominal | -0.942 | −0.021 | 0.303 | 0.003 | −1.566,−0.432 |
| Other | -0.249 | −0.015 | 0.323 | 0.432 | −0.900,0.331 |
| WBC (×10^9^/L) | 0.038 | 0.001 | 0.014 | 0.004 | 0.010,0.069 |
| PLT (×10^9^/L) | −0.001 | 0.000 | 0.001 | 0.288 | −0.002,0.001 |
| ln[PCT (ng/mL)] | 0.290 | 0.009 | 0.044 | 0.001 | 0.196,0.415 |
| LAC (mmol/L) | 0.006 | 0.000 | 0.035 | 0.848 | −0.069,0.073 |
| D-dimer (μg/mL) | 0.069 | 0.002 | 0.018 | 0.001 | 0.038–0.113 |
| MAP (mmHg) | 0.001 | 0.000 | 0.005 | 0.774 | −0.008,0.011 |

Model 2: Clinical baseline model plus SF

| Variable | Bootstrap | | | | |
| --- | --- | --- | --- | --- | --- |
|  | B | Bias | SE | *P* | BCa 95% CI |
| Age | 0.016 | 0.001 | 0.008 | 0.051 | −0.002,0.037 |
| Sex | 0.074 | 0.011 | 0.202 | 0.725 | −0.327,0.497 |
| Comorbidities |  |  |  |  |  |
| Hypertension | 0.124 | 0.011 | 0.215 | 0.558 | −0.330,0.602 |
| Diabetes mellitus | −0.141 | −0.007 | 0.201 | 0.468 | −0.515,0.246 |
| Coronary artery disease | 0.105 | 0.006 | 0.219 | 0.606 | −0.376,0.561 |
| Stroke | −0.558 | −0.015 | 0.225 | 0.015 | −1.013,−0.172 |
| Site of infection |  |  |  |  |  |
| Respiratory tract | −0.497 | −0.017 | 0.259 | 0.044 | −0.969,−0.042 |
| Urinary tract | 0.353 | 0.008 | 0.221 | 0.092 | −0.075,0.799 |
| Intra-abdominal | −0.972 | −0.037 | 0.343 | 0.002 | −1.677,−0.391 |
| Other | −0.364 | −0.019 | 0.387 | 0.330 | −1.139,0.289 |
| WBC (×10^9^/L) | 0.027 | 0.001 | 0.014 | 0.053 | −0.005,0.059 |
| PLT (×10^9^/L) | 0.000 | 0.000 | 0.001 | 0.875 | −0.001,0.002 |
| ln[PCT (ng/mL)] | 0.292 | 0.008 | 0.051 | 0.001 | 0.195,0.412 |
| LAC (mmol/L) | −0.019 | −0.001 | 0.036 | 0.564 | −0.085,0.046 |
| D-dimer (μg/mL) | 0.062 | 0.002 | 0.021 | 0.001 | 0.019,0.112 |
| MAP (mmHg) | 0.004 | 0.000 | 0.006 | 0.425 | −0.006,0.015 |
| SF (per 100 ng/mL) | 0.908 | 0.028 | 0.088 | 0.001 | 0.717,1.195 |
